# Understanding biological systems through the lens of data

**DOI:** 10.1186/s12918-017-0450-0

**Published:** 2017-09-21

**Authors:** Yong Wang, Xiang-Sun Zhang, Luonan Chen

**Affiliations:** 1 Academy of Mathematics and Systems Science, National Center for Mathematics and Interdisciplinary Sciences, University of Chinese Academy of Sciences, Chinese Academy of Sciences, Beijing, 100190 China; 2 Key Laboratory of Systems Biology, SIBS-Novo Nordisk Translational Research Centre for PreDiabetes, Shanghai Institutes for Biological Sciences, Chinese Academy of Sciences, Shanghai, 200031 China

## Abstract

A report of the 10th International Conference on Systems Biology (ISB2016), 19–22 August, Weihai, China.

## Background

The last ten years have witnessed a revolution in the generation of high throughput data in almost every aspect of biological and biomedical sciences. Examples include personalized genomics to electronic health records. Future biomedical science and clinical care will rely heavily on the data analysis and modelling, i.e., generation of meaningful knowledge from the integration of distinct sources. Along with the pressing need of strong quantitative approaches to deal with the big data, this energetic interdisciplinary field has kept attracting excellent scientists and making significant progresses to convert the biological data to fundamental insights in biology and medicine. Our International Conference on Computational Systems Biology (ISB), launched ten years ago [1–4], continues to serve as a high-quality platform and brought many researchers and students to freely exchange ideas. The 10th International Conference on Computational Systems Biology (ISB2016) was successfully organized by Chinese Academy of Sciences and Shandong University. We believe that the joint efforts of societies, funding agencies, research institutes, and universities will further push the development of computational methodologies, algorithms, and software in big data era.

## Meeting report

A three-day international conference on Computational and Systems Biology was held in in Weihai, China, August 19–22. More than 200 researchers including mathematicians, engineers, physicians, and biologists from China mainland, United States, Hong Kong, Taiwan, Japan, Korea enjoyed both academic exchanges and beautiful beaches in Weihai. To celebrate the ten-year anniversary, ISB2016 organized the beach soccer match in the early morning before conference. Many faculty members and students joined the match and showed their skills and truly young spirit.

Sixty-seven submissions to ISB2016 cover wide range of computational systems biology. Moreover, the reviewers from the Program Committee of ISB2016 selected 10 papers to be recommended for a special issue in BMC Systems Biology after significant extension of their original submission. Each submission has been peer reviewed and evaluated by three independent reviewers on the quality, originality, soundness, and significance of its contributions. Here we focus on some of the highlights of the meeting by categorizing and briefly introducing these selected papers.

We are currently generating massive data sets. Especially sequencing data is growing astronomically. New algorithms for data analysis and integrations are in pressing need. In this issue, Gan et al. propose a framework called *mimvec* to analyze the human phenome by making use of the state-of-the-art deep learning technique in natural language processing. They converted 24,061 records in the Online Mendelian Inheritance in Man (OMIM) database to low-dimensional vectors. They demonstrated that the vector presentation not only effectively enabled classification of phenotype records against gene ones, but also succeeded in discriminating diseases of different inheritance styles and different mechanisms.

He et al. present an accelerated parallel implementation of ARACNE (GPU-ARACNE). By taking advantage of multi-level parallelism and the Compute Unified Device Architecture (CUDA) parallel kernel-call library, GPU-ARACNE successfully parallelizes a serial algorithm and simplifies the user experience from multi-step operations to one step. Using public datasets on comparable hardware configurations, they showed that GPU-ARACNE is faster than previous implementations and is able to reconstruct equally valid gene regulatory networks.

Tian et al. present an initialization-and-refinement framework for inferring direct PPI networks from AP-MS data, in which an initial network is first generated with existing scoring methods and then a refined network is constructed by the application of indirect association removal methods. Experimental results on several real AP-MS data sets show that their method is capable of identifying more direct interactions than traditional scoring methods.

Zhang et al. use maximum likelihood and Bayesian inference method to establish phylogenetic trees. Multi-chain Markov chain Monte Carlo sampling method is used to select optimal phylogenetic tree, resolving local optimum problem. Fu et al. utilized probabilistic graphical model to integrate genetic interaction and protein interaction data and infers exquisitely detailed pathway structure. The results indicate that the new method performs better in predicting signalling pathways than existing models.

Liu et al. proposed a mathematical model for the Delta-Notch dependent boundary formation in the Drosophila large intestine in order to better interpret related experimental findings of this biological phenomenon.

Sun et al. proposed a novel network-based probabilistic generative model, NetGen, to perform the functional enrichment analysis. An additional protein-protein interaction (PPI) network was explicitly used to assist the identification of significantly enriched GO terms. NetGen achieved a superior performance than the existing methods in the simulation studies. NetGen has been implemented in the R package CopTea publicly available at GitHub (http://github.com/wulingyun/CopTea/).

Shi et al. presents a computational model for Notch1 signalling pathway in glioma cells. Based on the bifurcation analysis of the model, they show that how the glioma cell fate decisions are modulated by both trans-activation and cis-inhibition mediated by the Fringe protein, providing insight into the design and control principles of the Notch signalling system and the gliomas.

Guo et al. proposed a novel hybrid method to combine several heuristic strategies to study HP model on 3D lattice. The results indicate that our hybrid method can predict protein structure more accurately and efficiently. Furthermore, it serves as a useful tools to probe the protein stability on 3D lattice and provides some biological insights.

Gao et al. studied the different states of influenza A by the method of dynamical network biomarkers. Through establishing protein dynamical network biomarkers of influenza A virus protein, a composite index is ultimately obtained to forecast influenza A pandemic outbreak.
